# Reducing Stress and Anxiety in First-Year Undergraduates Through Biofeedback-Assisted Relaxation Training

**DOI:** 10.7759/cureus.48200

**Published:** 2023-11-03

**Authors:** V R Abhinaya Ravada, Kakarla V M Sai Lahari, Havilah Twinkle Reddipogu, Lakshmi Prasanna Vuyyuru, Chandana Konda, Mohammed Jaffer Pinjar

**Affiliations:** 1 Psychiatry, Great Eastern Medical School and Hospital (GEMS) Ragolu, Srikakulam, IND; 2 Physiology, Maharaja’s Institute of Medical Sciences, Vizianagaram, IND; 3 Physiology, All India Institute of Medical Sciences, Deoghar, IND

**Keywords:** medical education, undergraduate students, anxiety, stress, biofeedback

## Abstract

Background*:* College students face many stressors daily, often resulting in psychological challenges such as heightened anxiety and depression. Biofeedback is becoming aware of and learning to modify the body’s physiological functions. This study aimed to evaluate the efficacy of biofeedback relaxation training in mitigating stress and anxiety levels among medical undergraduates.

Methods*: *This pretest-posttest control group design study was conducted following the ethics committee’s approval. The study selected first-year MBBS students based on their elevated stress and anxiety scores and divided them into intervention and control groups. The study involved subjecting the intervention group to 10 sessions of biofeedback relaxation training, while the control group was not given any treatment. Stress and anxiety levels were assessed before and after the intervention of 10 to 12 weeks using the Perceived Stress Scale-14 and Cognitive Test Anxiety Scale.

Results*: *The study involved 93 students, with 47 of them in the intervention group and the remaining 46 in the control group. After biofeedback, the intervention group showed significantly lower stress and anxiety scores than the control group.

Conclusion*:* Biofeedback training effectively reduced stress and anxiety levels among undergraduate medical students. Biofeedback techniques can be implemented in college settings to support student mental wellness.

## Introduction

College students encounter a range of stressors on a daily basis, including adapting to the demands of higher education, handling a heavier academic workload, navigating interpersonal challenges with faculty and peers, and managing financial pressures [1]. Academic stress in college students encompasses a variety of elements. These include dealing with environmental stressors like interactions with peers and faculty, managing academically-related pressures such as exams and assignments, and their psychological reactions to these stressors, such as anxiety and depression [2]. Academic stress leads to mental discomfort among students, often stemming from the fear of potential failure or underperformance. Key sources of this stress include exams, an overload of assignments, issues with managing time, strained relationships, competition among peers, and overly high expectations. Stress can lead to decreased attentional resources, potentially hampering distinguishing between relevant and irrelevant information. This situation may increase a student’s distractibility, resulting in subpar exam performance, ineffective social interactions, and poor time management [3]. Numerous research studies indicate that students often display signs of mental distress, including depression, severe anxiety, phobias, physical discomfort, irritability, crying episodes, and diminished interest in their studies. Martin and Smith’s work also reveals a direct correlation between stress and thoughts of suicide. A failure in academic performance has been linked to a five times higher risk of a suicide attempt [4]. Research indicates that due to the stressors mentioned earlier, college students are more vulnerable to mental health issues such as anxiety, depression, thoughts of suicide, difficulties in decision-making, and higher dropout rates. During these stressful periods, students may experience various emotional disruptions, which can sometimes drive them to adopt unhealthy coping mechanisms, self-medication, or seek beneficial psychological therapies [5]. Clinical biofeedback has been identified as a beneficial tool for managing symptoms of illness and enhancing overall health and wellness by providing training in stress management [6]. Biofeedback is gaining awareness of the body’s physiological functions. Training in biofeedback aids individuals in altering their physiological activity, enhancing their health and performance [7]. The study aimed to investigate the effects of biofeedback therapy as an intervention technique on undergraduate medical students’ stress and anxiety levels. This article was previously presented as a poster at the 13th Annual Conference of Andhra Pradesh Medical Educators Association-2023 on October 6th, 2023.

## Materials and methods

The study was conducted as a pretest-posttest control group design research project after obtaining ethical approval from the institutional ethics committee. The study population comprised first-year MBBS students selected through purposive sampling from the teaching institute. After obtaining informed consent, students were administered tools like Perceived Stress Scale-14 (PSS-14) and Cognitive Test Anxiety Scale (CTAS) before formative assessment. Among them, students with heightened stress and anxiety scores were chosen and divided into two groups. The study involved two groups: the intervention group, which received biofeedback relaxation training (BFRT), and the control group, which received no treatment. The BFRT group underwent 10 sessions spaced over 10-12 weeks. After completing the biofeedback sessions, both groups were evaluated using PSS-14 and CTAS before the next formative assessment, and their scores were compared.

Compared to conventional cognitive-behavioral therapy (CBT) techniques for stress reduction, biofeedback intervention training is a relatively recent development. This self-regulation technique empowers individuals to consciously control bodily processes previously believed to be beyond their control. Specialized equipment is used to translate physiological signals into significant visual cues. Participants receive feedback on a computer screen or other display, which assists them in gaining mastery over their physiological responses. Biofeedback is a technique that empowers individuals to actively participate in modifying their physiology, leading to enhanced physical, mental, emotional, and spiritual well-being. Stress management training using clinical biofeedback is an effective approach to managing symptoms of illness and promoting overall health and well-being [8]. The PSS-14 is a 14-item questionnaire that measures how individuals perceive situations as stressful. The scale proposed by Cohen et al. assesses the degree to which situations are perceived as unpredictable, uncontrollable, and intense [9]. The questions are created to assess the present levels of stress experienced, and the scale has a Cronbach alpha value of 0.70, indicating its reliability. The scale, which includes both positively and negatively worded items, asks participants to rate their feelings on a five-point scale ranging from 0 (never) to 4 (very often) concerning their experiences in the last four weeks. It is advisable to administer the scale after four to six weeks to ensure accurate results, considering its temporal nature.

The CTAS is a questionnaire of 24 items that aims to evaluate the cognitive aspects of test anxiety throughout the entire learning test cycle, including the preparation and performance phases. Higher scores on the scale indicate greater levels of cognitive test anxiety. The responses obtained can be used to categorize learners into low, moderate, or high levels of cognitive test anxiety [10]. Statistical analysis: The data collected was entered into a Microsoft Excel sheet and analyzed using IBM Corp. Released in 2015. IBM SPSS Statistics for Windows, Version 23.0. Armonk, NY: IBM Corp. Descriptive statistics were used to obtain frequencies, means, and percentages with a 95% confidence interval. Inferential statistics were performed using a t-test, and a P-value of 0.05 was considered statistically significant.

## Results

The study comprised 93 students. The rationale for this sample size is convenient. Forty-seven (50.5%) students belonged to the intervention group, for whom biofeedback was done, 46 (49.4%) students constituted the control group. Thirty-three (35.4%) were male students, and 60 (64.5%) were female students. Assessing by T-test, the mean PSS score and mean CTAS score prior to biofeedback among the intervention group were found to be 29.87 (standard deviation (SD): 4.96 ) and 56.00 (SD: 6.70), respectively. In the counterpart control group, scores were 30.07 (SD: 4.8) and 56.02 (SD: 6.35) respectively as given in Tables 1-2.

**Table 1:** PSS-14 scores in the study population PSS: Perceived Stress Scale-14; N: number of sample; SD: standard deviation

Group	N (%)	Baseline Mean (SD)	End-of-Study Mean (SD)	Difference Mean (SD)	P-value
Intervention	47 (50.5%)	29.87 (4.96)	14.83 (4.19)	15.04 (4.2)	0.001
Control	46 (49.4%)	30.07 (4.8)	25.07 (4.26)	5.0 (3.85)	

**Table 2:** CTAS scores in the study population CTAS: Cognitive Test Anxiety Scale

Group	N (%)	Baseline Mean (SD)	End-of-study Mean (SD)	Difference Mean (SD)	P-value
Intervention	47 (50.5%)	56.00 (6.70)	33.79 (5.8)	22.21 (5.61)	0.001
Control	46 (49.4%)	56.02 (6.35)	45.89 (8.25)	10.13 (5.12)	

After biofeedback, the mean PSS and CTAS scores among the intervention group were 14.83 (SD: 4.19) and 33.79 (SD: 5.8), respectively. At the end of the study, control group scores were 25.07 (SD: 4.26) and 45.89 (SD: 8.25), respectively. On paired t-test analysis, the P-value was 0.001, which was statistically significant. The mean difference between the PSS and CTAS scores with biofeedback among the intervention group was 15.04 (SD: 4.2) and 22.21 (SD: 5.61), respectively. The control group’s mean difference scores were 5.0 (SD: 3.85) and 10.13 (SD: 5.12) as given in the Tables 1-2.

In the intervention group, there were 17 males (36.1%) and 30 females (63.8%). At baseline, the mean PSS score for males was 29.45 (SD: 4.41) and for females was 30.25 (SD: 5.09). The mean CTAS score for males was 56 (SD: 6.94) and for females was 55.97 (SD: 6.3). By the end of the study, the mean PSS score had decreased to 19.61 (SD: 6.42) for males and 20.05 (SD: 6.81) for females. The mean CTAS score had also decreased to 39.55 (SD: 9.79) for males and 39.90 (SD: 9.15) for females. The P-value for both genders was 0.001, indicating a statistically significant change in both PSS and CTAS scores from the baseline to the end of the study as per Table 3.

**Table 3:** Gender, PSS-14, and CTAS scores in the intervention group PSS: Perceived Stress Scale-14; CTAS: Cognitive Test Anxiety Scale

Group	N (%)	Baseline Mean (SD)	End-of-study Mean (SD)	P-value
Males	17 (36.1%)	PSS- 29.45 (4.41) CTAS- 56 (6.94)	PSS- 19.61 (6.42) CTAS- 39.55 (9.79)	0.001
Females	30 (63.8%)	PSS- 30.25 (5.09) CTAS- 55.97 (6.3)	PSS- 20.05 (6.81) CTAS- 39.90 (9.15)	0.001

## Discussion

The research objective was to evaluate the efficiency of biofeedback relaxation training in reducing stress and anxiety levels among students. Biofeedback is a method that utilizes specialized equipment to provide individuals with information about their physiological functions to control these functions in response. It can enhance health and performance by tracking physiological changes related to thoughts, emotions, and behavior. The term “biofeedback” is composed of two basic words: “bio,” which refers to biological therapy aspects, and “feedback,” implying a stimulus that promotes internal behavioral change in an individual [11]. These findings were evident from the study. When an individual places more emphasis on success than failure and believes positively in their ability to manage stress, a stressor can be seen as a challenge. However, if the stressor is perceived as a threat, it can lead to anxiety and negatively impact cognitive function and academic performance. The stress response can be detrimental in situations focusing on failures and their consequences and a lack of self-belief in managing stress. The statement emphasizes that stress can have both positive and negative impacts, whereas anxiety is only associated with negative effects. By utilizing biofeedback, it is possible to transform anxiety-inducing threat responses into more positive challenge responses [12].

When students experience prolonged stress, their everyday balance of autonomic functions tends to lean toward a state dominated by the sympathetic nervous system, reducing parasympathetic activity. This consistent decrease in parasympathetic activity can impair the body’s ability to regulate physiological functions in response to external stressors. Over time, students may become susceptible to physical and mental health disorders if the balance does not shift back from being predominantly sympathetic. Various techniques such as yoga, meditation, biofeedback, CBT, and life-skills training have been employed to manage stress [13]. In this study, 47 students participated in the biofeedback intervention. No baseline differences in score measures between the intervention and control groups were observed. The mean PSS scores decreased better with biofeedback compared to the control group in this study (Table 1). These findings were consistent with the Reddy’s study [6]. Students typically employ two primary strategies to handle academic stress. The initial strategy is problem-focused coping, where students identify and analyze the primary stressors to assess if any changes can be implemented. The second approach is emotion-focused coping, where students seek emotional assistance from their loved ones and peers. Social assistance and emotional aid are crucial in utilizing emotional coping as a behavioral and cognitive strategy to manage stress effectively. Cognitive therapeutic approaches and relaxation therapy can also effectively aid students in managing stress. Relaxation techniques can soothe muscles that may become tense due to stress [14].

The findings of this study align with the research conducted by Zwan et al., Biofeedback Heart Rate Variability training, which strengthens the homeostatic system’s reflexes and enhances the parasympathetic system’s functioning, is central to this approach [15]. It is vital to recognize students experiencing high stress levels and aim to enhance their psycho-social well-being. The data from this study stage suggest a general decrease in student stress levels following the intervention. A similar result was found in a study by Reddy involving 31 students [6]. While comprehending stressors can contribute to heightened self-awareness, enhancing personal resources to manage stress fosters psychological well-being and cultivates adaptability. The training aims to alleviate stress by focusing on the response or reaction to stress rather than the stressor itself. Van Daele et al. conducted a study that showed comparable outcomes with significant differences observed among undergraduate students who underwent biofeedback training and those who did not [16]. The current study conducted interventions for each participant over 10-12 weeks. A significant decrease in stress scores could be linked to the duration of the program, as previous research by Steffen et al. has shown that physiological changes may occur within three weeks [17]. Ratanasiripong et al. conducted a biofeedback intervention targeting stress reduction in public health students, yielding similar outcomes. Significant reductions in stress levels were observed following a four-week intervention program. Additionally, this training calms the body and enhances students’ ability to cope with academic stress [18].

Examinations are a critical and unavoidable aspect of any professional curriculum and often cause stress and exhaustion for students. Many students feel burdened due to the high expectations from parents, the tendency to compare themselves with others, and the pressure exerted by their peers [19]. Medical professionals often experience exam anxiety, which can cause physical, cognitive, and behavioral symptoms before and during exams. Addressing this issue is crucial and requires significant attention. Medical students face significant stress during and before examination periods due to various factors, including a rigorous curriculum, a highly competitive environment, the pressure to achieve high grades, the need to comprehend a vast syllabus quickly, and financial issues [20]. A moderate exam anxiety level can benefit students as it demonstrates their concern and keeps them engaged, attentive, and concentrated. Excessive worry can demotivate students and negatively impact their performance if not properly managed [21]. Lifestyle issues like insufficient rest, lack of time, poor nutrition, and inadequate physical activity management can lead to exam anxiety. Anxiety is one of the most significant threats to academic performance, with numerous studies indicating its harmful effects [22].

The change in scores was calculated by subtracting the end-of-study scores from the baseline scores. The findings showed a substantial improvement (P-0.001) for the group that received the intervention compared to the control group. According to the study conducted by Deckro et al., the intervention group reported a P-value of less than 0.018, which aligns with our findings [23]. A significant decrease in mean CTAS score by 22.2 was observed after biofeedback in the intervention group (Table 2) before formative assessment. The analysis of post-intervention differences between the biofeedback and control groups revealed that the biofeedback intervention significantly reduced anxiety levels among participants. Ratanasiripong et al. conducted a study whose findings are consistent with this result [18]. This study further highlighted the significant impact on stress levels in the intervention group. A decrease in both stress and anxiety levels among both males and females was observed (Table 3) in the study in the intervention group. No significant impact was observed regarding gender on the change of PSS-14 and CTAS scores with biofeedback.

It is important to approach the findings of the study with caution because of the limitations it has. The participants were self-selected, which may limit the generalizability of the results to the entire college campus. Additionally, the sample comprised a higher proportion of females, indicating a need to actively recruit more male participants for better gender representation in future studies. Further studies must include other college students to increase the generalizability. In future studies, it is important to consider including postgraduates and examining undergraduate and postgraduate students separately, as stress levels and health conditions can vary across different age groups. Obtaining a comprehensive understanding of the issue of academic stress among postgraduate students requires insights from not only the students themselves but also the faculty and administration. Developing an effective intervention module for students is worth considering variables such as personality factors and a history of psychiatric illness. Conducting a longitudinal study with annual follow-ups can provide further insights into the long-term impact of biofeedback intervention on participants’ mental health.

## Conclusions

Biofeedback training has proven to be an effective method for significantly reducing stress and anxiety levels in graduate students over 10 weeks. Cognitive impairments, leading to memory, attention, and concentration deficits, can negatively impact students’ overall well-being. As such, educational institutions must foster a balanced academic environment that promotes better learning and caters to students’ personal needs. It is also essential to note that for the positive changes from the training to have a lasting effect, participants must continue practicing the relaxation exercises after the training has ended. Research supports using biofeedback techniques to reduce stress, which can be effectively integrated into college settings to support student mental health.
